# Acute morphine activates satellite glial cells and up-regulates IL-1β in dorsal root ganglia in mice via matrix metalloprotease-9

**DOI:** 10.1186/1744-8069-8-18

**Published:** 2012-03-22

**Authors:** Temugin Berta, Tong Liu, Yen-Chin Liu, Zhen-Zhong Xu, Ru-Rong Ji

**Affiliations:** 1 Sensory Plasticity Laboratory, Pain Research Center, Department of Anesthesiology, Brigham and Women's Hospital, Harvard Medical School, Boston, MA 02115, USA; 2 Department of Anesthesiology, College of Medicine, National Cheng Kung University, Tainan city, Taiwan

## Abstract

**Background:**

Activation of spinal cord glial cells such as microglia and astrocytes has been shown to regulate chronic opioid-induced antinociceptive tolerance and hyperalgesia, due to spinal up-regulation of the proinflammatory cytokines such as interleukin-1 beta (IL-1β). Matrix metalloprotease-9 (MMP-9) has been implicated in IL-1β activation in neuropathic pain. However, it is unclear whether acute opioid treatment can activate glial cells in the peripheral nervous system. We examined acute morphine-induced activation of satellite glial cells (SGCs) and up-regulation of IL-1β in dorsal root ganglia (DRGs), and further investigated the involvement of MMP-9 in these opioid-induced peripheral changes.

**Results:**

Subcutaneous morphine injection (10 mg/kg) induced robust peripheral glial responses, as evidenced by increased GFAP expression in DRGs but not in spinal cords. The acute morphine-induced GFAP expression is transient, peaking at 2 h and declining after 3 h. Acute morphine treatment also increased IL-1β immunoreactivity in SGCs and IL-1β activation in DRGs. MMP-9 and GFAP are expressed in DRG neurons and SGCs, respectively. Confocal analysis revealed a close proximity of MMP-9 and GFAP immunostaining. Importantly, morphine-induced DRG up-regulation of GFAP expression and IL-1β activation was abolished after *Mmp9 *deletion or naloxone pre-treatment. Finally, intrathecal injections of IL-1β-selective siRNA not only reduced DRG IL-1β expression but also prolonged acute morphine-induced analgesia.

**Conclusions:**

Acute morphine induces opioid receptors- and MMP-9-dependent up-regulation of GFAP expression and IL-1β activation in SGCs of DRGs. MMP-9 could mask and shorten morphine analgesia via peripheral neuron-glial interactions. Targeting peripheral glial activation might prolong acute opioid analgesia.

## Background

Mounting evidence indicates that activation of spinal cord glial cells such as microglia and astrocytes plays a crucial role in the pathogenesis of chronic pain [1-7]. In particular, chronic opioid exposure induces profound changes in spinal cord microglia and astrocytes [8-10]. Upon activation spinal glial cells produce multiple proinflammatory cytokines such as TNF-α, IL-1β, and IL-6 to antagonize morphine analgesia and promote morphine tolerance [11-14], via sensitization of spinal cord dorsal horn neurons [15-17]. For example, IL-1β has been shown to counteract opioid-induced analgesia following both chronic and acute administration of morphine [18]. While intrathecal injection of the inteurleukin-1 receptor (IL-1R) antagonist potentiates acute morphine analgesia [11], intrathecal administration of IL-1β induces heat hyperalgesia [15]. Of interest genetic polymorphism of IL-1R antagonist has been implicated in the variation in postoperative morphine consumption [19].

Our previous study has shown that matrix metalloproteinase 9 (MMP-9) plays an important role in neuroinflammation and neuropathic pain development in part through the activation (active cleavage) of IL-1β [20]. MMP-9 has also been shown to regulate the phenotype and proliferation of peripheral [21] and central [20] glial cells. In a parallel study, we also showed that acute morphine induced a rapid up-regulation of MMP-9 in primary sensory neurons in the dorsal root ganglia (DRGs), masking morphine-induced analgesia (Liu et al., unpublished data). However, the molecular and cellular mechanisms by which MMP-9 suppresses/shortens acute opioid analgesia are unclear.

Satellite glial cells (SGCs) are peripheral glial cells and form a continuous layer around primary sensory neurons within DRGs and trigeminal ganglia (TGs). SGCs have been shown to regulate neuronal homeostasis and neurotransmission in DRGs and TGs [22]. Increasing evidence suggests that SGCs exhibit marked morphological and biochemical changes following peripheral nerve injury and inflammation [23-27]. Peripheral inflammation suppresses inward rectifying potassium currents and increases IL-1β expression in SGCs of TG, leading to neuronal firing and trigeminal pain [28,29]. Chemokine signaling in DRG SGCs and neurons was shown to regulate chronic opioid-induced hyperalgesia [30]. However, it is unclear whether and how SGCs are activated in DRGs following acute opioid treatment. Our data demonstrated that a single subcutaneous morphine injection could induce MMP-9 and opioid receptors-dependent activation of SGCs and IL-1β in DRGs, highlighting the importance of peripheral neuronal-satellite glial interactions for the control of acute opioid analgesia.

## Results

### Subcutaneous morphine induces GFAP expression in DRGs but not in spinal cords

**Figure 1:**
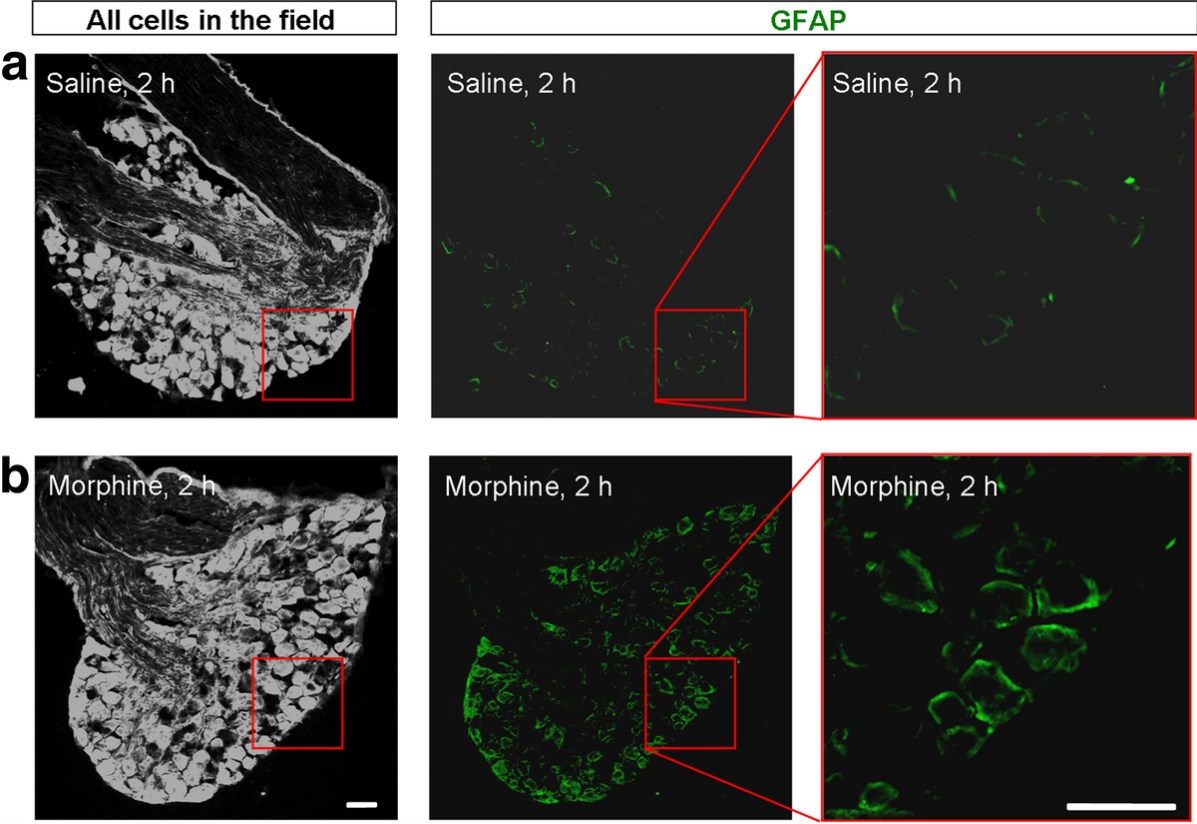
**Subcutaneous morphine increases GFAP immunoreactivity in DRG SGCs**. **(A, B) **GFAP immunoreactivity in DRGs from saline (A) or morphine (B, 10 mg/kg, s.c.) treated mice at 2 h after the injections. Left panels are images with high contrast showing all DRG cells in an optic field. Middle and right panels are low and high magnification images of GFAP staining. Note there is a marked increase in GFAP staining after acute morphine treatment. Scales, 50 μm.

The first question we addressed is whether acute morphine-induced acute analgesia is associated with peripheral glial responses in DRGs. We used GFAP as a marker for SGCs and performed immunohistochemistry to examine GFAP expression in DRGs, 2 h after saline and morphine injection when morphine analgesia declines. In DRGs of saline-treated control mice, only weak GFAP staining was observed (Figure 1A). Strikingly, the intensity of GFAP staining was markedly increased in DRGs of morphine-treated mice (Figure 1B).

**Figure 2:**
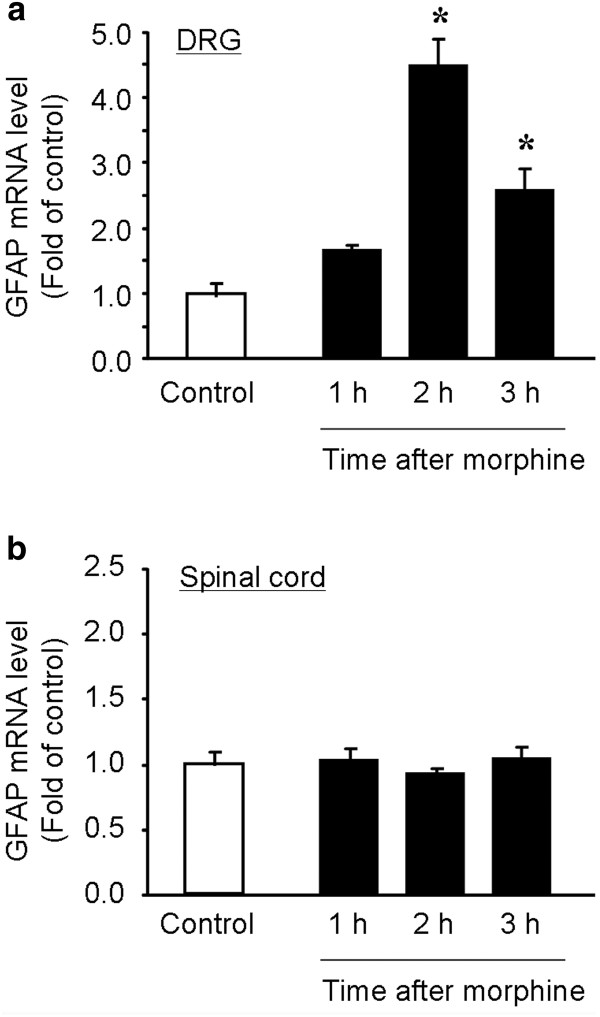
**Subcutaneous morphine induces GFAP mRNA expression in DRGs but not in spinal cords**. **(A, B) **Real-time RT-PCR showing the time course of GFAP expression in lumbar DRGs (A) and spinal cord dorsal horns (B) after subcutaneous morphine injection (10 mg/kg, s.c.). Note that acute morphine only increases GFAP mRNA expression in DRGs but not in spinal cord dorsal horns. The data are expressed as fold of naïve controls. *P < 0.05, vs. control, ANOVA followed by Bonferroni post hoc test, n = 4 mice.

Next, we performed qPCR analysis to quantify the GFAP mRNA levels in DRGs and spinal cords at different times of morphine injection. Acute morphine increased GFAP mRNA expression in DRGs [F(3,12) = 30.9, *P *< 0.0001, One-Way ANOVA], which peaked at 2 h (4.5 fold of control, *P *< 0.05, n = 4) and declined but still remained elevated at 3 h (2.6 fold of control, *P *< 0.05, n = 4) (Figure 2A). In contrast, acute morphine did not change GFAP mRNA expression in spinal cord dorsal horns at all the time points we examined (F(3,12) = 0.51, *P *= 0.682, One-Way ANOVA] (Figure 2B). Thus, it appears that acute morphine only causes reaction of peripheral glia (SGCs) but not of central glia, since GFAP is a hallmark for astrocytes in the CNS.

### MMP-9 is required for subcutaneous morphine-induced GFAP expression in DRG SGCs

**Figure 3:**
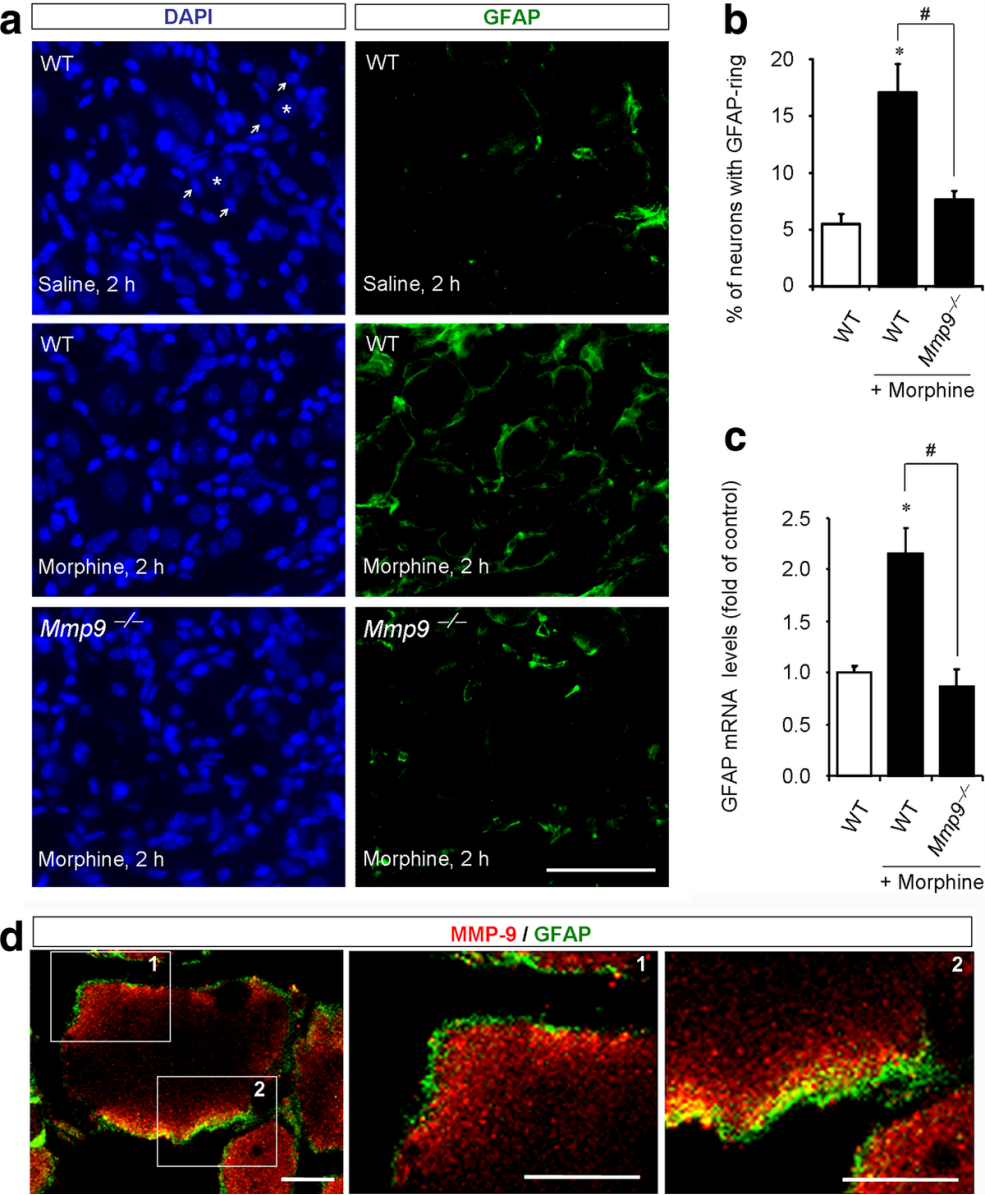
**MMP-9 is required for subcutaneous morphine-induced GFAP expression in SGCs of DRGs**. **(A) **Left panels: DAPI nuclei staining of all cells in DRG sections of saline (up panel) and morphine (2 h, middle and low panels) treated wild-type (WT) mice and morphine-treated *Mmp9 *knockout mice (Mmp9^-/-^, low panel). Stars and arrows indicate neurons (no staining) and SGCs, respectively. Note that *Mmp9 *knockout mice show no changes in DRG cell numbers. Right panels: Immunohistochemistry showing GFAP-labeled SGCs in DAPI-stained DRG sections. Scale, 50 μm. **(B) **Percentage of DRG neurons with GFAP-labeled ring. Note that morphine-induced GFAP increase at 2 h is abrogated in *Mmp9 *knockout mice. *P < 0.05, compared to WT control; ^#^*P *< 0.05 compared to morphine/WT, ANOVA followed by Bonferroni post hoc test, n = 6 mice. **(C) **GFAP mRNA expression in DRGs revealed by real time RT-PCR analysis. Note that morphine-induced GFAP mRNA expression (2 h) in WT mice is abolished in *Mmp9 *knockout mice. *P < 0.05, compared to WT control; ^#^*P *< 0.05 compared to morphine/WT; ANOVA followed by Bonferroni post hoc test, n = 4. **(D) **Confocal images showing doubles staining of MMP-9 and GFAP in DRG sections of WT mice 2 h after morphine treatment. Square 1 and 2 are enlarged in middle and right panels. Note there is close proximity but not overlap of MMP-9 and GFAP staining. Scales, 10 μm.

GFAP-positive SGCs were found to form ring-like staining around 5.5% DRG neurons of saline-treated mice (Figure 3A, B). This percentage increased to 17.1 at 2 h after morphine injection (Figure 3A, B; *P *< 0.05, n = 6). To determine the contribution of MMP-9 to morphine-elicited SGC reaction, we examined GFAP expression in *Mmp9 *null mice (*Mmp9*^-/-^). Compared to wild-type control mice, morphine-induced increase in ring-like staining of GFAP was abolished in *Mmp9*^-/- ^mice, and only 7.6% DRG neurons showed GFAP-ring (Figure 3A, B, P **<**0.05, n = 6). Furthermore, qPCR analysis revealed that morphine-induced DRG increase in GFAP mRNA expression was also abrogated in *Mmp9*^-/- ^mice compared to that of the wild-type mice (Figure 3C; 0.9 fold of vehicle, *P ***<**0.05, n = 4). However, the basal expression levels of GFAP mRNA in the wild-type and *Mmp9*^-/- ^mice were very similar (*P ***= **0.2468, Mann-Whitney test, n = 4). Together, these data suggest that MMP-9 is essential for GFAP up-regulation in DRG-SGCs following acute morphine treatment.

In a parallel study, we demonstrated MMP-9 up-regulation in DRG neurons after acute morphine treatment (Liu et al., unpublished data). MMP-9 could be secreted to extracellular space to trigger the reaction of surrounding SGCs via neuronal-glial interaction. Double staining of MMP-9 and GFAP and confocal microscopy examination revealed that MMP-9 and GFAP-labeled structures were in very close proximity but not overlapped (Figure 3D), providing a structural base for MMP-9-initiated neuronal-glial interaction in DRGs.

### Subcutaneous morphine induces IL-1β activation in DRG SGCs via MMP-9

**Figure 4:**
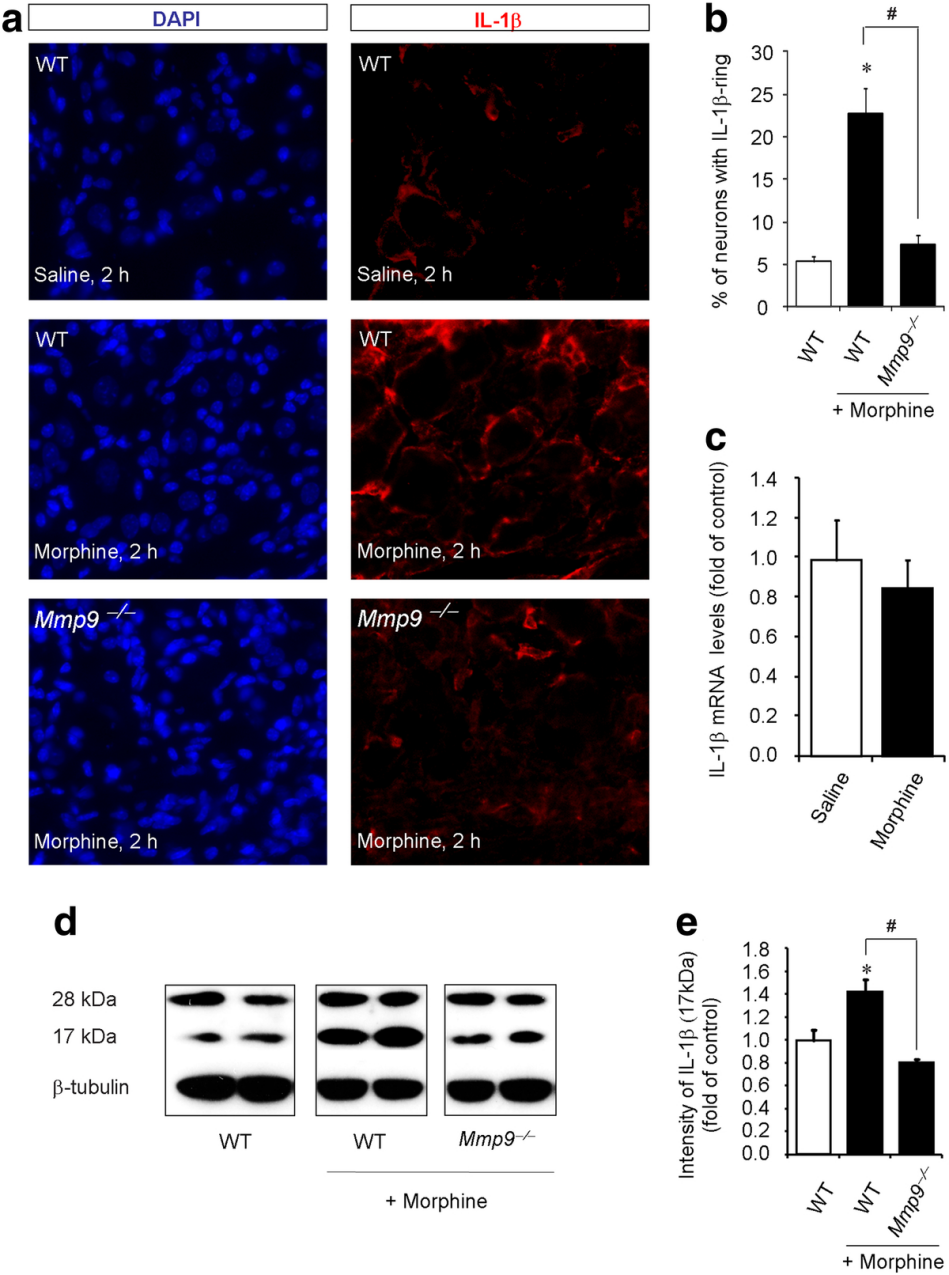
**MMP-9 is required for subcutaneous morphine-induced IL-**1β **activation in DRGs**. **(A) **DAPI nuclei staining (left panels) and IL-1β immunostaining (right panels) in DRG sections of saline (upper panels) and morphine (middle panels) treated wild-type (WT) mice and morphine-treated *Mmp9 *knockout mice (*Mmp9*^-/-^, lower panels). Scale, 50 μm. **(B) **Percentage of DRG neurons with IL-1β-labeled ring. Note that morphine-induced IL-1β increase is abrogated in the *Mmp9 *knockout mice. *P < 0.05, compared to WT control; ^# ^*P *< 0.05 compared to morphine/WT, ANOVA followed by Bonferroni post hoc test, n = 6 mice. **(C) **Real-time RT-PCR analysis showing IL-1β mRNA expression in DRGs of saline and morphine treated mice (10 mg/kg, s.c., 2 h). *P *> 0.05, Mann-Whitney test, n = 4. **(D) **Western blotting showing non-mature (28 kDa) and mature/active (17 kDa) bands of IL-1β in DRGs of WT and *Mmp9*^-/- ^mice with or without morphine treatment (10 mg/kg, s.c., 2 h). Note that the 28 kDa bands were unaltered after morphine treatment. **(E) **Intensity of the active IL-1β (17 kDa) bands. Note that morphine-induced activation of IL-1β is abrogated in *Mmp9*^-/-^mice. *P **<**0.05, compared to WT control; ^#^*P ***<**0.05 compared to morphine/WT, ANOVA followed by Bonferroni post hoc test, n = 4 mice.

Chronic morphine but not acute morphine was shown to increase IL-1β levels in the spinal cord [11]. We set out to test if acute morphine would induce IL-1β expression and activation in DRGs. Low level of IL-1β expression was observed in DRGs of saline-treated wild-type animals: only 5.4% DRG neurons were surrounded by IL-1β-expressing staining in the control animals. However, this percentage increased to 22.7 in morphine-treated wild-type mice (Figure 4A, B; *P *< 0.05, n = 6). Notably, this increase was abrogated in *Mmp9*^-/- ^mice, and only 7.4% DRG neurons were surrounded by IL-1β-expressing satellite cells (Figure 4A, B; *P *< 0.05, n = 6). However, acute morphine did not alter the expression of the IL-1β mRNA (Figure 4C).

Next, we conducted Western blot analysis to check the activation of IL-1β in DRGs after morphine treatment. Morphine produced no significant change of the 28 kDa band of IL-1β, the non-mature form of IL-1β (Figure 4D). In contrast, morphine induced a significant increase of the 17 kDa band of IL-1β, the active form of IL-1β, in DRGs of wild-type mice (Figure 4D, E, 1.4 fold of control, *P *< 0.05, n = 4). Notably, this increase of the active form of IL-1β was completely abolished in *Mmp9*^-/- ^mice (Figure 4D, E).

**Figure 5:**
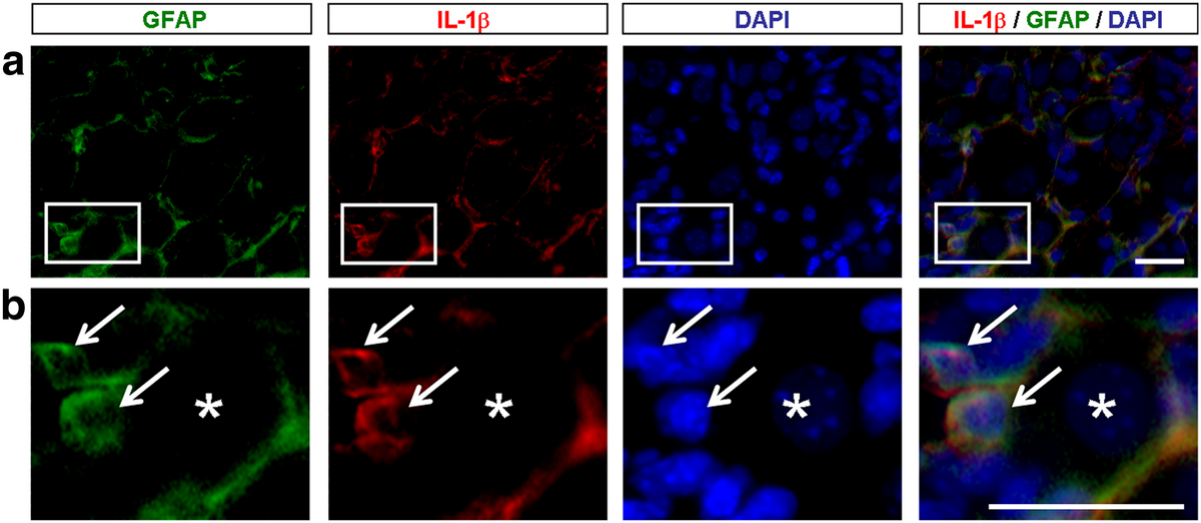
**Co-localization of GFAP and IL-1β in SGCs of DRGs**. **(A) **Triple staining showing co-localization of GFAP (green), IL-1β (red), and DAPI (blue) in DRG-SGCs of morphine (2 h)-treated mice. Star and arrows indicate neurons and satellite glia, respectively. **(B) **High magnification images of boxes in A. Scales, 20 μm.

To determine the cellular localization of IL-1β in DRGs, we carried out triple staining for GFAP, IL-1β, and DAPI (nuclear marker). We found a clear co-localization of GFAP and IL1β in cytoplasm of SGCs of morphine-treated mice (Figure 5). Together, our results suggest that MMP-9 is required for the morphine-induced IL-1β activation in SGCs.

### Intrathecal opioids increase GFAP expression and IL-1β activation in DRGs but not in spinal cords

**Figure 6:**
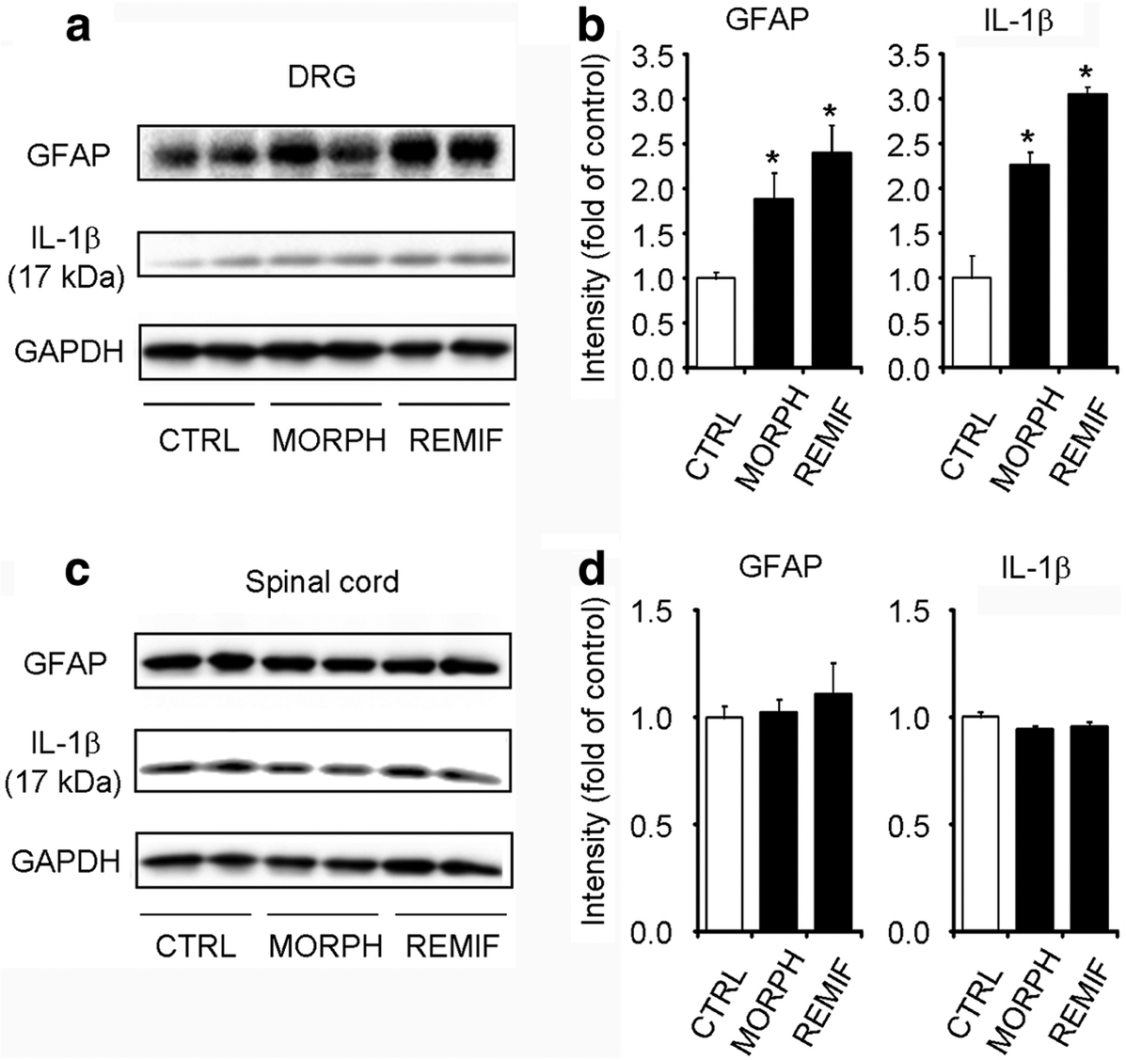
**Intrathecal injection of morphine and remifentanil increases GFAP expression and IL-1β activation in DRGs but not in spinal cords**. **(A) **Western blotting showing the expression of GFAP and IL-1β (17 kDa cleaved form) in DRGs after intrathecal injection of morphine (10 nmol, 2 h) and remifentanil (1 nmol, 2 h). **(B) **Quantification of GFAP and IL1β (17 kDa) bands in DRGs. *P < 0.05, compared to saline control; ANOVA followed by Bonferroni post hoc test, n = 4 mice. **(C) **Western blotting showing the expression of GFAP and IL-1β (17 kDa) in spinal cord dorsal horns after intrathecal injection of morphine (10 nmol, 2 h) and remifentanil (1 nmol, 2 h). **(D) **Quantification of GFAP and IL-1β bands in spinal cord dorsal horns. Note there is no change in spinal GFAP expression and IL-1β activation after morphine treatment. *P < 0.05, compared to saline control; ANOVA followed by Bonferroni post hoc test, n = 4 mice. CTRL, vehicle control; MORPH, morphine; REMIF, remifentanil.

To determine whether morphine would directly act on DRG cells to produce the observed effects, we intrathecally injected morphine or remifentanil, a potent fast-acting synthetic opioid. Single intrathecal injection of morphine (10 nmol) and remifentanil (1 noml) elicited significant increases in GFAP levels [(F(2,9) = 13.1, *P *= 0.002, One-Way ANOVA] and IL1β (17 kDa) levels [(F(2,9) = 8.6, *P *= 0.008, One-Way ANOVA] in DRGs (Figure 6A). In contrast, intrathecal morphine and remifentanil failed to induce GFAP and IL-1β (17 kDa) expression in spinal cord dorsal horns (Figure 6B). These results indicate that DRGs are primary target for acute opioid-induced GFAP expression and IL-1β activation.

### Intrathecal injection of IL-1β-targeting siRNA prolongs morphine analgesia

**Figure 7:**
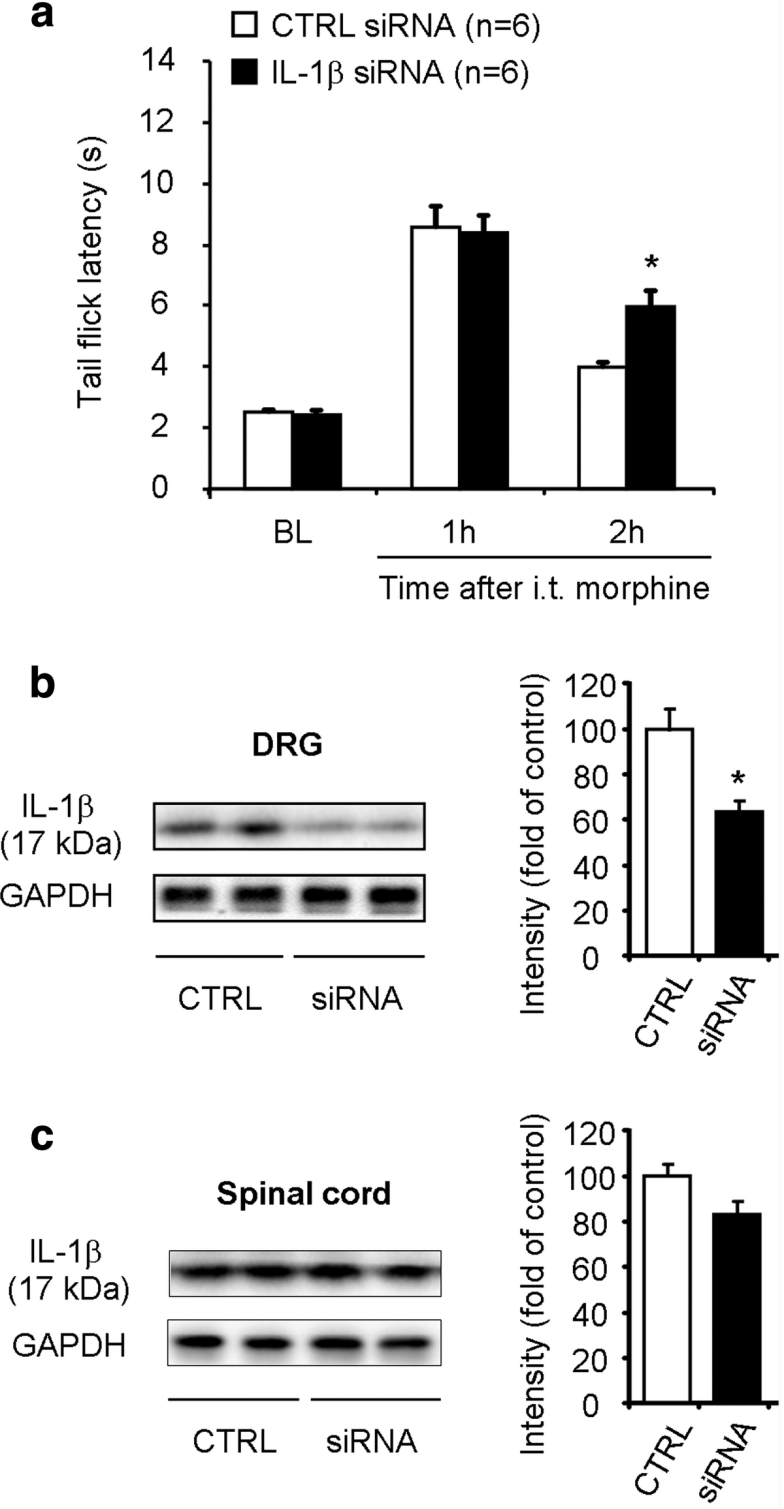
**Intrathecal injections of IL-1β siRNA prolong morphine analgesia and reduce IL-1β expression in DRGs**. **(A) **Acute morphine-induced analgesia is prolonged after intrathecal injections of IL-1β siRNA (3 μg, 24 and 2 h prior to morphine injection). Morphine analgesia was tested at 1 h and 2 h after the morphine injection (10 mg/kg, s.c.). *P **<**0.05, compared to non-targeting control (CTRL) siRNA, Two-Way ANOVA followed by Bonferroni post hoc test, n = 6 mice. **(B, C) **Western blotting analysis showing IL-1β expression (17 kDa) in DRGs (B) and spinal cord dorsal horns (C) after intrathecal injections of IL-1β siRNA (siRNA) or control siRNA. *P **<**0.05, compared to control, Mann-Whitney test, n = 6 mice.

IL-1β antagonist has been shown to potentiate acute morphine analgesia and reduce morphine tolerance [11,31]. We further evaluated the role of IL-1β in acute opioid analgesia using a different strategy to knock down DRG-IL-1β expression via intrathecal route. Compared to non-targeting control siRNA, IL-1β siRNA treatment significantly increased tail-flick latency at 2 h (Figure 7A, P < 0.05, Bonferroni post hoc test, n = 6) but not at 1 h (Figure 7A, P > 0.05). It is suggested that IL-1β siRNA treatment could prolong opioid analgesia.

We also examined the knockdown effects of IL-1β siRNA on IL-1β expression in DRG and spinal cord tissues collected 2 h after morphine injection. Notably, morphine-induced IL-1β expression in DRGs was reduced by 40% after intrathecal IL-1β siRNA treatment, compared with control siRNA (Figure 7B, P **<**0.05, Mann-Whitney test, n = 6). However, the same IL-1β siRNA treatment (i.t.) did not produce significant reduction of IL-1β expression in spinal cord dorsal horns at 2 h after morphine injection (Figure 7C, P > 0.05, Mann-Whitney test, n = 6). One possible explanation for the differential knockdown of DRG vs. spinal cord IL-1β by intrathecal siRNA treatment is that DRG IL-1β is inducible after morphine treatment, therefore, more sensitive to siRNA treatment, as shown in our previous study [32,33]. In contrast, knockdown of constitutive IL-1β expression in the spinal cord may require high doses and additional injections of siRNA. Since the IL-1β siRNA treatment only reduced DRG IL-1β, which is expressed in SGCs, we hypothesize that IL-1β expressed by peripheral DRG glia (SGCs) plays a role in masking/diminishing morphine-induced analgesia.

### Morphine induces GFAP up-regulation and IL-1β activation in DRG SGCs via opioid receptors

**Figure 8:**
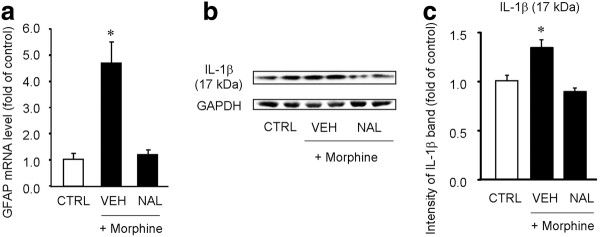
**Naloxone blocks subcutaneous morphine-induced GFAP expression and IL-1β activation in DRGs**. **(A) **Real-time RT-PCR showing GFAP mRNA expression in DRGs after subcutaneous morphine injection (10 mg/kg, 2 h) with or without naloxone pre-treatment (10 mg/kg, 15 min prior to the morphine injection). Note that morphine-induced GFAP expression is completely blocked by naloxone. The data are expressed as fold of controls. *P < 0.05, vs. control, ANOVA followed by Bonferroni post hoc test, n = 4 mice. **(B) **Western blotting showing the expression of active form of IL-1β (17 kDa) in DRGs after subcutaneous morphine with or without naloxone pre-treatment. **(C) **Intensity of IL-1β bands (17 kDa) in DRGs. Note that morphine-induced IL-1β activation is completely abolished by naloxone. *P < 0.05, compared to saline control, ANOVA followed by Bonferroni post hoc test, n = 4. CTRL, control; VEH, vehicle; NAL, naloxone; MORPH, morphine.

Morphine could induce SGC activation via opioid receptors-dependent and independent mechanisms, such as activation of toll-like receptors [34]. To address this issue, we investigated the effects of the opioid receptor antagonist naloxone on the morphine-induced GFAP expression and IL-1β activation. Pre-treatment of naloxone (10 mg/kg, s.c.), 10 min prior to the morphine injection, completely blocked the morphine-evoked GFAP mRNA expression (Figure 8A, P **<**0.05, One-Way ANOVA) and IL-1β activation (Figure 8B, C, P **<**0.05, One-Way ANOVA) in DRGs.

## Discussion

**Figure 9:**
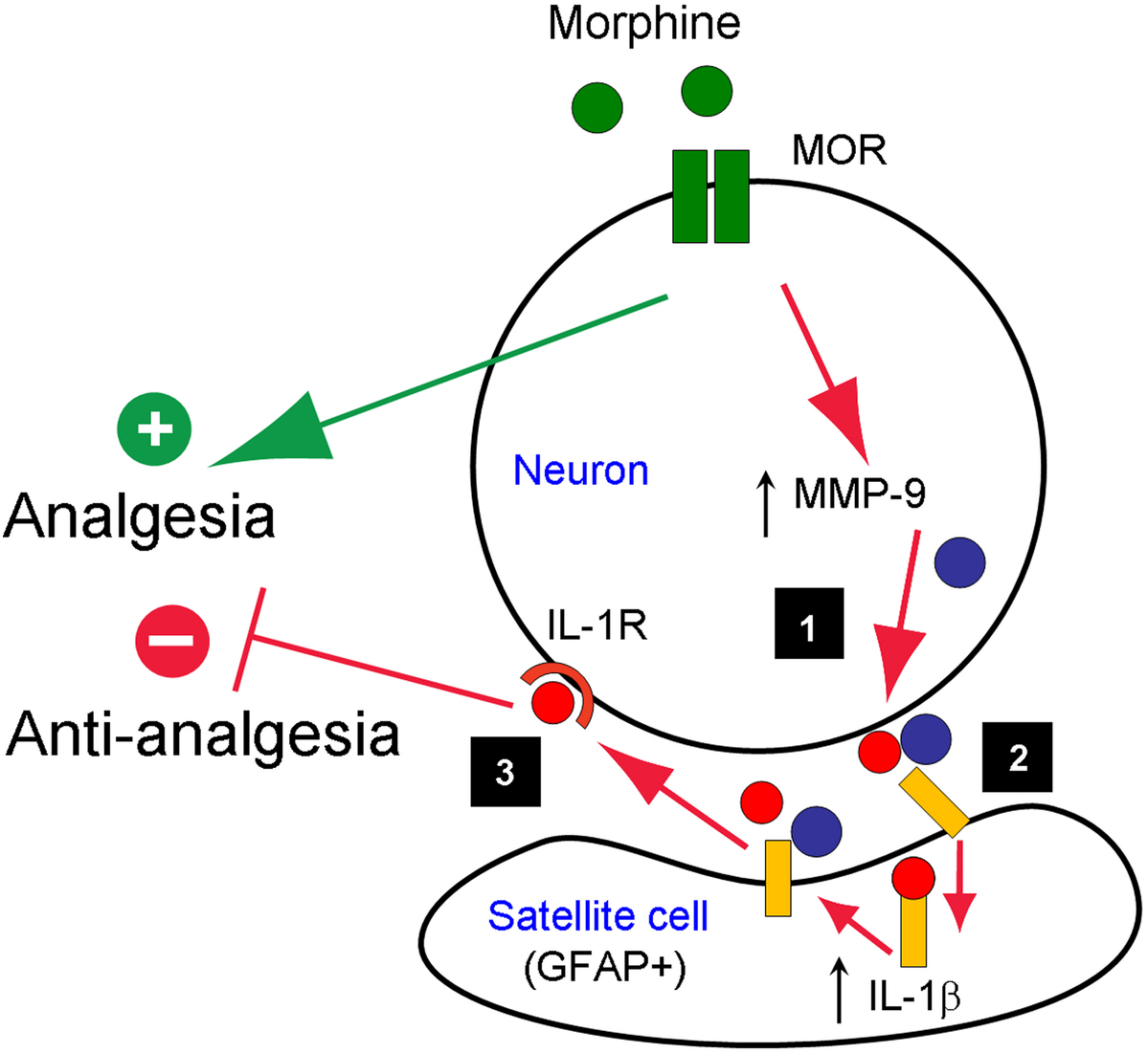
**Schematic of the working hypothesis illustrating how acute morphine induces SGCs activation in DRGs to mask morphine analgesia**. Acute morphine produces analgesia via μ opioid receptors (MOR). Acute morphine also induces MMP-9 up-regulation in DRG neurons via MOR (Step-1). MMP-9 is secreted in extracellular space, inducing glial reaction and IL-1β cleavage (Step-2). The activated (cleaved) IL-1β binds IL-1 receptors in neurons, in a paracrine or autocrine manner, and results in increasing excitability of nociceptive neurons to mask and shorten morphine analgesia (Step-3).

In the present study, together with another study in an accompanying paper (Liu et al., unpublished data), we provided several lines of evidence that support an important role of DRG satellite glial cells (SGCs) in masking and shortening opioid analgesia, via neuron-glial interactions in DRGs (Figure 9). (1) Subcutaneous or intrathecal opioid (morphine and remifentanil) induced rapid and transient MMP-9 expression (1-3 h) in DRG neurons but not in the spinal cord (Liu et al., unpublished data). (2) Subcutaneous or intrathecal opioid induced GFAP expression (at 2 h) in DRG SGCs but not in the spinal cord (Figures 1, 2, and 6). (3) Subcutaneous or intrathecal opioid induced IL-1β activation (at 2 h) in DRG SGCs but not in spinal cords (Figures 4 and 6). (4) The morphine-induced SGC activation (GFAP expression and IL-1β activation) in DRGs was abolished after *Mmp9 *deletion and naloxone pre-treatment (Figures 3, 4, and 8). (5) Acute morphine-induced analgesia was enhanced and prolonged after *Mmp9 *deletion or by MMP-9 inhibition via intrathecal route (Liu et al., unpublished data). (6) Intrathecal treatment of IL-1β siRNA (using a mild protocol with 2 injections) produced IL-1β knockdown in DRGs (but not in spinal cords) and also prolonged morphine-elicited analgesia (Figure 7). Based on these findings, we postulate that neuronal MMP-9 expression and subsequent MMP-9 release following acute morphine treatment triggers the activation of SGCs (IL-1β activation), diminishing the analgesic efficacy of opioid (Figure 9).

To the best of our knowledge, this is the first report showing that acute morphine could induce satellite glial activation in DRGs. We found significant increases in both mRNA and protein levels of the satellite glial marker GFAP after a single subcutaneous injection of morphine (Figures 1 and 2), indicating a peripheral glial reaction by acute morphine. In sharp contrast, previous studies have focused on chronic morphine treatment and central glial activation. For example, chronic morphine induced up-regulation of the microglial markers CD11b and IBA1 and the astrocyte marker GFAP in the spinal cord [8,9,12,35,36]. In particular, chronic morphine activates p38 MAP kinase in spinal cord microglia to promote morphine tolerance [10,36-38]. However, we failed to detect increases in the expression of spinal glial markers such as the astrocyte marker GFAP (Figures 2 and 6) and the microglial marker IBA-1 (data not shown) after single subcutaneous and intrathecal injections of morphine, suggesting that acute morphine primarily induces peripheral glial responses in DRGs.

SGCs in the DRG are tightly associated with sensory neurons via gap junction; and gap junction communication between SGCs and neurons is greatly enhanced in persistent pain conditions [39,40]. Neuron-glial interactions in DRGs may involve purinergic signaling [41,42]. After nerve injury, chronic inflammation, and DRG compression, SGCs exhibit robust up-regulation of GFAP [43-45]. Nerve injury-induced SGC reaction depends on neuronal activity and local inflammation [23,46]. Our data showed that morphine-induced GFAP induction in SGCs required MMP-9 (Figure 3). As an extracellular matrix protein, MMP-9 could be released from DRG neurons following morphine stimulation and retain in extracellular space between neurons and satellite cells. MMP-9 may cleave extracellular matrix proteins to induce satellite glial reaction and GFAP up-regulation (Figure 8). Of note MMP-9 immunostaining in neurons had very close proximity to GFAP immunostaining in SGCs (Figure 3D). MMP-9 has been shown to regulate phenotypic remodeling and proliferation of peripheral glial cells such as Schwann cells via signaling of IGF-1, ErbB receptors, and ERK [21].

In particular, our data demonstrated that MMP-9 could drive IL-1β activation after acute morphine. Acute morphine induced IL-1β activation in the DRG but not in the spinal cord (Figures 4 and 6). Similarly, a previous study failed to show IL-1β increase either in lumbar spinal cord or in CSF after acute morphine injection [11]. As previously reported [32,47,48], we observed IL-1β expression by DRG SGCs. However, we should not rule out the possibility that IL-1β could also be expressed in neurons by testing different IL-1β antibodies [32,49]. Primary sensory neurons particularly nociceptors express IL-1 receptors, and notably, activation of these receptors by IL-1β can rapidly activate nociceptors to generate action potentials and elicit pain hypersensitivity [50]. It appears that IL-1β increases the excitability of sensory neurons via increasing sodium currents and decreasing potassium currents [48,50,51]. Importantly, intrathecal interleukin-1 receptor antagonist potentiates both intrathecal and systemic morphine-induced analgesia [11,31]. Since IL-1β activation following acute morphine was primarily found in the DRG, we postulate that intrathecal interleukin-1 receptor antagonist may also enhance opioid analgesia by blocking IL-1β signaling in DRGs. This notion is strongly supported by our siRNA experiment, showing that morphine analgesia was prolonged after IL-1β knockdown in DRGs by selective siRNA treatment (Figure 7).

Importantly, *Mmp9 *deletion resulted in a complete loss of morphine-induced IL-1β activation in the DRG (Figure 4). IL-1β is activated via cleavage from its precursor (≈ 31 kDa) and this activation is not only mediated by caspase-1 (i.e. IL-1β converting enzyme) but also mediated by other enzymes such as trypsin, elastase, collagenase, and cathepsin G, and MMP-9 [52-54]. MMP-9 can cause direct activation of IL-1β (generation of 17 kDa form) in a cell-free system [53]. Notably, caspase-1-deficient mice can still produce bioactive IL-1β and mediate IL-1β-dependent reactions under several pathological conditions [55]. Consistently, nerve injury-induced DRG-IL-1β activation is abolished in *Mmp9*^-/- ^mice, whereas intrathecal MMP-9 increases IL-1β activation in DRGs [32]. However, we should not exclude the possibility that at very high concentrations MMP-9 may also cause IL-1β degradation and inactivation via direct cleavage of IL-1β at different sites [56].

In summary, opioids are still the most used analgesics for the treatment of moderate to severe pain, despite their well-known side effects. Increasing evidence shows that spinal cord glial cells and proinflammatory cytokines contribute to chronic opioid-induced antinociceptive tolerance and hyperalgesia via neuronal-glial interactions in the spinal cord [12,13]. We have demonstrated that acute morphine also induces peripheral satellite glial cell (SGC) activation in the DRG, which is triggered by the protease MMP-9. Morphine-induced MMP-9 up-regulation in DRG neurons could mask morphine analgesia via neuronal-glial interactions. Specifically, MMP-9 caused IL-1β activation, leading to hyperexcitability of primary sensory neurons to mask morphine analgesia (Figure 9). Thus, opioids not only produce excitatory effects in the spinal cord via postsynaptic [57,58] and presynaptic mechanisms [59], they could further elicit excitatory effects at DRG level via MMP-9-triggered peripheral neuronalglial interactions. Targeting peripheral neuronal-glial interactions in DRGs may prolong the analgesic efficacy of opioids.

## Methods

### Animals

All experiments were performed in accordance with the guidelines of the National Institutes of Health and the International Association for the Study of Pain. All animals were used under Harvard Medical School Animal Care institutional guidelines. Adult male mice (25-35 g) were used for behavioral and biochemical studies, including CD1 mice (Charles River Laboratories, Wilmington, MA), *Mmp9 *konckout (*Mmp9*^-/-^) mice with FVB background, and FVB control wild-type mice. *Mmp9 *knockout (KO) mice and FVB wild-type mice were obtained from Jackson Laboratories (The Jackson Laboratory, Bar Harbor, ME). The KO mice are viable, fertile, and maintained in FVB background for more than 5 generations. Mice that are homozygous null for the *Mmp9 *gene were used in this study. As previously described, they do not show any difference in overall weight and behavior compared with wild-type mice [32]. Animals were housed in a 12 h light/dark room with access to food and water *ad libitum*.

### Drugs and administration

We purchased morphine sulphate and remifentanil from Hospira (Lake Forest, IL) and naloxone form Sigma (St. Louis, MO). Drugs were freshly prepared in saline and administered subcutaneously in the lower back (10 mg/kg for morphine and naloxone) or intrathecally (10 nmol for morphine and 1 nmol for remifentanil). The IL-1β (GCUCCGAGAUGAACAACAA) and control siRNAs (GACUUCGCGGGACACAUGA) were purchased from Dharmacon (Dharmacon, Inc., Chicago, IL). siRNA was dissolved in RNase-free water at the concentration of 1 μg/μl as stock solution, and mixed with polyethyleneimine (PEI, Fermentas Inc., Glen Burnie, MD), 10 min before injection, to increase cell membrane penetration and reduce the degradation. PEI was dissolved in 5% glucose, and 1 μg of siRNA was mixed with 0.18 μl of PEI. siRNA (3 μg) was intrathecally injected 24 and 2 h before the morphine injection. Of note, agents with different chemical properties have been shown to affect DRG cells via intrathecal route, including small molecules such as MAP kinase and MMP-9 inhibitors [32,60] and large molecules such as growth factors and peptides [61,62], as well as antisense oligodeoxynucleotides [63,64] and siRNAs [33,65]. Intrathecal injection of p38 inhibitor can rapidly inhibit p38 activation in DRG neurons within half hour [66]. Thus, intrathecal delivery of siRNA should affect cells both in DRGs and spinal cords.

### Behavioral test

All animals (n = 6 mice per group) were habituated to testing environment for at least 2 days before baseline testing. Morphine analgesia was evaluated by measuring the tail-flick latencies in hot water [67]. Briefly, tail-flick test was performed by gently holding the mouse wrapped with a terry towel and kept tail exposed. Then one third of the length of the tail was immersed into the 52°C hot water, and the response latency was defined as removal of the whole tail from the water. A maximum cut-off value of 10 seconds was set to avoid thermal injury.

### Immunohistochemistry

Two hours after morphine or vehicle (saline) injection, animals (n = 6 mice per group) were terminally anesthetized with isoflurane and perfused through the ascending aorta with PBS, followed by 4% paraformaldehyde with 1.5% picric acid in 0.16 M PB. After the perfusion, DRGs (L4/L5) were removed and postfixed in the same fixative overnight. DRG sections (12 μm) were cut in a cryostat and processed for immunofluorescence. All the sections were blocked with 10% goat serum, and incubated over night at 4°C with the primary antibodies against GFAP (mouse, 1:2000, Millipore, Billerica, MA), MMP-9 (1:1000, rabbit, Millipore), or IL-1β (1:1000, rabbit, Millipore; 1:500, goat, R&D System, Minneapolis, MN) with 5% goat serum. The sections were then incubated for 1 h at room temperature with Cy3- or FITC-conjugated secondary antibody (1:400, Jackson Immunolab, West Grove, PA) with 1% goat serum. DAPI (4',6-diamidino-2-phenylindole; Sigma) staining was used to determine the cell nuclei. For double immunofluorescence, sections were incubated with a mixture of polyclonal and monoclonal primary antibodies followed by a mixture of FITC- and CY3-congugated secondary antibodies [68]. The stained sections were examined under a Nikon fluorescence microscopy, and images were captured with a CCD camera (SPOT, Diagnostic Instruments). To obtain high-resolution images, confocal images were captured from some DRG section with a Zeiss LSM 510 META upright confocal microscope. All images were analyzed with NIH Image software or Adobe Photoshop.

### Western blot

Animals (n = 4-6 mice per group) were terminally anesthetized with isoflurane at 1, 2, and 3 h after morphine injection and transcardially perfused with PBS. DRGs (L4/L5) and spinal cord segments (dorsal part of L4-L5) were rapidly removed and homogenized in a lysis buffer containing a cocktail of protease inhibitors and phosphatase inhibitors [69]. The protein concentrations were determined by BCA Protein Assay (Pierce Biotechnology, Inc., Rockford, IL). Twenty micrograms of proteins were loaded for each lane and separated on SDS-PAGE gel (4-15%, Bio-Rad). After the transfer, the blots were incubated overnight at 4°C with polyclonal antibody against GFAP (mouse, 1:1000, Millipore) or IL-1β (1:1000, rabbit, Millipore). For loading control, the blots were probed with β-tubulin or GAPDH antibody (mouse, respectively 1:2000 and 1:20000, Sigma).

### Quantitative real-time PCR (qPCR)

Animals (n = 4 per group) were terminally anesthetized with isoflurane at 1, 2, and 3 h after morphine injection. DRGs (L4/L5) and spinal cord dorsal horn segments (L4-L5) were rapidly dissected and total RNA from each animal was isolated using RNeasy Plus Mini kit (Qiagen, Valencia, CA). One microgram of RNA was reverse transcribed (RT) using Omniscript reverse transcriptase according to the protocol of the manufacturer (Qiagen). The sequences for the forward and reverse primers of GFAP and GAPDH are included in Table 1. SYBR-green qPCR analyses were performed using the Opticon real-time PCR Detection System (Bio-Rad, Hercules, CA) as described previously [70]. Briefly, qPCR amplification reactions contained RT products, 7.5 μl of 2X iQSYBR-green mix (Bio-Rad), 300 nM of forward and reverse primers completed with nanopure water for a final volume of 15 μl. The thermal cycling conditions were: 3 min at 95°C for the polymerase activation, 45 cycles of 10 s at 95°C for denaturation, and 30 s at 60°C for annealing and extension, followed by a DNA dissociation curve for the determination of the amplicon specificity. Analyses were carried out in triplicates and included the different primer efficiencies obtained by a standard curve method.

**Table 1:** Sequences of the primer sets for RT-PCR

Target Gene	Forward Primers	Reverse Primers	Genbank No.
**GFAP**	**GAATCGCTGGAGGAGGAGAT**	**GCCACTGCCTCGTATTGAGT**	NM010277

**GAPDH**	**TCCATGACAACTTTGGCATTG**	**CAGTCTTCTGGGTGGCAGTGA**	XM001473623

### Statistical analyses

All results are presented as means ± SEM. Differences between means were tested for significance using Mann-Whitney test, 1-way or 2-way ANOVA followed by Bonferroni post hoc test. Significance was determined at a level of *P *< 0.05.

## Competing interests

The authors declare that they have no competing interests.

## Authors' contributions

TB, YCL, and ZZX performed biochemical and histochemical experiments; TL performed behavioral testing; RRJ, TB, and TL designed the experiments; and RRJ and TB wrote the manuscript. All authors read and approved the final manuscript.

## References

[B1] Tsuda M Kuboyama K Inoue T Nagata K Tozaki-Saitoh H Inoue K Behavioral phenotypes of mice lacking purinergic P2X4 receptors in acute and chronic pain assays Mol Pain 2009 5 28 10.1186/1744-8069-5-28 19515262 PMC2704200

[B2] Inoue K Tsuda M Microglia and neuropathic pain Glia 2009 57 1469 1479 10.1002/glia.20871 19306358

[B3] Gao YJ Ji RR Targeting astrocyte signaling for chronic pain Neurotherapeutics 2010 7 482 493 10.1016/j.nurt.2010.05.016 20880510 PMC2950097

[B4] Milligan ED Watkins LR Pathological and protective roles of glia in chronic pain Nat Rev Neurosci 2009 10 23 36 10.1038/nrn2533 19096368 PMC2752436

[B5] McMahon SB Malcangio M Current challenges in glia-pain biology Neuron 2009 64 46 54 10.1016/j.neuron.2009.09.033 19840548

[B6] Ren K Dubner R Interactions between the immune and nervous systems in pain Nat Med 2010 16 1267 1276 10.1038/nm.2234 20948535 PMC3077564

[B7] Katsura H Activation of Src-family kinases in spinal microglia contributes to mechanical hypersensitivity after nerve injury 2006 10.1523/JNEUROSCI.1771-06.2006 PMC6674378 16928856

[B8] Song P Zhao ZQ The involvement of glial cells in the development of morphine tolerance Neurosci Res 2001 39 281 286 10.1016/S0168-0102(00)00226-1 11248367

[B9] Raghavendra V Rutkowski MD DeLeo JA The role of spinal neuroimmune activation in morphine tolerance/hyperalgesia in neuropathic and sham-operated rats J Neurosci 2002 22 9980 9989 12427855 10.1523/JNEUROSCI.22-22-09980.2002 PMC6757841

[B10] Wen YR Tan PH Cheng JK Liu YC Ji RR Microglia: a promising target for treating neuropathic and postoperative pain, and morphine tolerance J Formos Med Assoc 2011 110 487 494 10.1016/S0929-6646(11)60074-0 21783017 PMC3169792

[B11] Johnston IN Milligan ED Wieseler-Frank J Frank MG Zapata V Campisi J A role for proinflammatory cytokines and fractalkine in analgesia, tolerance, and subsequent pain facilitation induced by chronic intrathecal morphine J Neurosci 2004 24 7353 7365 10.1523/JNEUROSCI.1850-04.2004 15317861 PMC6729781

[B12] Watkins LR Hutchinson MR Johnston IN Maier SF Glia: novel counter-regulators of opioid analgesia Trends Neurosci 2005 28 661 669 10.1016/j.tins.2005.10.001 16246435

[B13] DeLeo JA Tanga FY Tawfik VL Neuroimmune activation and neuroinflammation in chronic pain and opioid tolerance/hyperalgesia Neuroscientist 2004 10 40 52 10.1177/1073858403259950 14987447

[B14] Ren K Torres R Role of interleukin-1beta during pain and inflammation Brain Res Rev 2009 60 57 64 10.1016/j.brainresrev.2008.12.020 19166877 PMC3076185

[B15] Kawasaki Y Zhang L Cheng JK Ji RR Cytokine mechanisms of central sensitization: distinct and overlapping role of interleukin-1beta, interleukin-6, and tumor necrosis factor-alpha in regulating synaptic and neuronal activity in the superficial spinal cord J Neurosci 2008 28 5189 5194 10.1523/JNEUROSCI.3338-07.2008 18480275 PMC2408767

[B16] Li J Xie W Zhang JM Baccei ML Peripheral nerve injury sensitizes neonatal dorsal horn neurons to tumor necrosis factor-alpha Mol Pain 2009 5 10 10.1186/1744-8069-5-10 19254372 PMC2657778

[B17] Zhang L Berta T Xu ZZ Liu T Park JY Ji RR TNF-alpha contributes to spinal cord synaptic plasticity and inflammatory pain: distinct role of TNF receptor subtypes 1 and 2 Pain 2011 152 419 427 10.1016/j.pain.2010.11.014 21159431 PMC3022092

[B18] Hutchinson MR Coats BD Lewis SS Zhang Y Sprunger DB Rezvani N Proinflammatory cytokines oppose opioid-induced acute and chronic analgesia Brain Behav Immun 2008 22 1178 1189 10.1016/j.bbi.2008.05.004 18599265 PMC2783238

[B19] Bessler H Shavit Y Mayburd E Smirnov G Beilin B Postoperative pain, morphine consumption, and genetic polymorphism of IL-1beta and IL-1 receptor antagonist Neurosci Lett 2006 404 154 158 10.1016/j.neulet.2006.05.030 16777324

[B20] Kawasaki Y Xu ZZ Wang X Park JY Zhuang ZY Tan PH Distinct roles of matrix metalloproteases in the early- and late-phase development of neuropathic pain Nat Med 2008 14 331 336 10.1038/nm1723 18264108 PMC2279180

[B21] Chattopadhyay S Shubayev VI MMP-9 controls Schwann cell proliferation and phenotypic remodeling via IGF-1 and ErbB receptor-mediated activation of MEK/ERK pathway Glia 2009 57 1316 1325 10.1002/glia.20851 19229995 PMC2713381

[B22] Hanani M Satellite glial cells in sensory ganglia: from form to function Brain Res Brain Res Rev 2005 48 457 476 15914252 10.1016/j.brainresrev.2004.09.001

[B23] Xie W Strong JA Zhang JM Early blockade of injured primary sensory afferents reduces glial cell activation in two rat neuropathic pain models Neuroscience 2009 160 847 857 10.1016/j.neuroscience.2009.03.016 19303429 PMC2777638

[B24] Dublin P Hanani M Satellite glial cells in sensory ganglia: their possible contribution to inflammatory pain Brain Behav Immun 2007 21 592 598 10.1016/j.bbi.2006.11.011 17222529

[B25] Dubovy P Klusakova I Svizenska I Brazda V Satellite glial cells express IL-6 and corresponding signal-transducing receptors in the dorsal root ganglia of rat neuropathic pain model Neuron Glia Biol 2010 6 73 83 10.1017/S1740925X10000074 20519054

[B26] Liang L Wang Z Lu N Yang J Zhang Y Zhao Z Involvement of nerve injury and activation of peripheral glial cells in tetanic sciatic stimulation-induced persistent pain in rats J Neurosci Res 2010 88 2899 2910 20544834 10.1002/jnr.22439

[B27] Zhang H Mei X Zhang P Ma C White FA Donnelly DF Altered functional properties of satellite glial cells in compressed spinal ganglia Glia 2009 57 1588 1599 10.1002/glia.20872 19330845 PMC2759416

[B28] Takeda M Tanimoto T Kadoi J Nasu M Takahashi M Kitagawa J Enhanced excitability of nociceptive trigeminal ganglion neurons by satellite glial cytokine following peripheral inflammation Pain 2007 129 155 166 10.1016/j.pain.2006.10.007 17127002

[B29] Takeda M Takahashi M Nasu M Matsumoto S Peripheral inflammation suppresses inward rectifying potassium currents of satellite glial cells in the trigeminal ganglia Pain 2011 152 2147 2156 10.1016/j.pain.2011.05.023 21680091

[B30] Wilson NM Jung H Ripsch MS Miller RJ White FA CXCR4 signaling mediates morphine-induced tactile hyperalgesia Brain Behav Immun 2011 25 565 573 10.1016/j.bbi.2010.12.014 21193025 PMC3039030

[B31] Hutchinson MR Coats BD Lewis SS Zhang Y Sprunger DB Rezvani N Proinflammatory cytokines oppose opioid-induced acute and chronic analgesia Brain Behav Immun 2008 22 1178 1189 10.1016/j.bbi.2008.05.004 18599265 PMC2783238

[B32] Kawasaki Y Xu ZZ Wang X Park JY Zhuang ZY Tan PH Distinct roles of matrix metalloproteases in the early- and late-phase development of neuropathic pain Nat Med 2008 14 331 336 10.1038/nm1723 18264108 PMC2279180

[B33] Tan PH Yang LC Ji RR Therapeutic potential of RNA interference in pain medicine Open Pain J 2009 2 57 63 10.2174/1876386300902010057 19966919 PMC2788313

[B34] Watkins LR Hutchinson MR Johnston IN Maier SF Glia: novel counter-regulators of opioid analgesia Trends Neurosci 2005 28 661 669 10.1016/j.tins.2005.10.001 16246435

[B35] Zhou D Chen ML Zhang YQ Zhao ZQ Involvement of spinal microglial P2X7 receptor in generation of tolerance to morphine analgesia in rats J Neurosci 2010 30 8042 8047 10.1523/JNEUROSCI.5377-09.2010 20534852 PMC6632682

[B36] Horvath RJ Landry RP Romero-Sandoval EA DeLeo JA Morphine tolerance attenuates the resolution of postoperative pain and enhances spinal microglial p38 and extracellular receptor kinase phosphorylation Neuroscience 2010 169 843 854 10.1016/j.neuroscience.2010.05.030 20493931 PMC2904400

[B37] Cui Y Chen Y Zhi JL Guo RX Feng JQ Chen PX Activation of p38 mitogen-activated protein kinase in spinal microglia mediates morphine antinociceptive tolerance Brain Res 2006 1069 235 243 10.1016/j.brainres.2005.11.066 16403466

[B38] Ji RR Targeting microglial purinergic signaling to improve morphine analgesia Pain 2010 150 377 378 10.1016/j.pain.2010.06.010 20584568 PMC2921463

[B39] Hanani M Huang TY Cherkas PS Ledda M Pannese E Glial cell plasticity in sensory ganglia induced by nerve damage Neuroscience 2002 114 279 283 10.1016/S0306-4522(02)00279-8 12204197

[B40] Dublin P Hanani M Satellite glial cells in sensory ganglia: their possible contribution to inflammatory pain Brain Behav Immun 2007 21 592 598 10.1016/j.bbi.2006.11.011 17222529

[B41] Chen Y Zhang X Wang C Li G Gu Y Huang LY Activation of P2X7 receptors in glial satellite cells reduces pain through downregulation of P2X3 receptors in nociceptive neurons Proc Natl Acad Sci USA 2008 105 16773 16778 10.1073/pnas.0801793105 18946042 PMC2575495

[B42] Zhang X Chen Y Wang C Huang LY Neuronal somatic ATP release triggers neuron-satellite glial cell communication in dorsal root ganglia Proc Natl Acad Sci USA 2007 104 9864 9869 10.1073/pnas.0611048104 17525149 PMC1887586

[B43] Takeda M Takahashi M Matsumoto S Contribution of the activation of satellite glia in sensory ganglia to pathological pain Neurosci Biobehav Rev 2009 33 784 792 10.1016/j.neubiorev.2008.12.005 19167424

[B44] Hu P Bembrick AL Keay KA McLachlan EM Immune cell involvement in dorsal root ganglia and spinal cord after chronic constriction or transection of the rat sciatic nerve Brain Behav Immun 2007 21 599 616 10.1016/j.bbi.2006.10.013 17187959

[B45] Zhang H Mei X Zhang P Ma C White FA Donnelly DF Altered functional properties of satellite glial cells in compressed spinal ganglia Glia 2009 57 1588 1599 10.1002/glia.20872 19330845 PMC2759416

[B46] Li JY Xie W Strong JA Guo QL Zhang JM Mechanical hypersensitivity, sympathetic sprouting, and glial activation are attenuated by local injection of corticosteroid near the lumbar ganglion in a rat model of neuropathic pain Reg Anesth Pain Med 2011 36 56 62 10.1097/AAP.0b013e318203087f 21455091 PMC3076946

[B47] Takeda M Takahashi M Matsumoto S Contribution of activated interleukin receptors in trigeminal ganglion neurons to hyperalgesia via satellite glial interleukin-1beta paracrine mechanism Brain Behav Immun 2008 22 1016 1023 10.1016/j.bbi.2008.03.004 18440198

[B48] Takeda M Tanimoto T Kadoi J Nasu M Takahashi M Kitagawa J Enhanced excitability of nociceptive trigeminal ganglion neurons by satellite glial cytokine following peripheral inflammation Pain 2007 129 155 166 10.1016/j.pain.2006.10.007 17127002

[B49] Copray JC Mantingh I Brouwer N Biber K Kust BM Liem RS Expression of interleukin-1 beta in rat dorsal root ganglia J Neuroimmunol 2001 118 203 211 10.1016/S0165-5728(01)00324-1 11498255

[B50] Binshtok AM Wang H Zimmermann K Amaya F Vardeh D Shi L Nociceptors are interleukin-1beta sensors J Neurosci 2008 28 14062 14073 10.1523/JNEUROSCI.3795-08.2008 19109489 PMC2690713

[B51] Takeda M Kitagawa J Takahashi M Matsumoto S Activation of interleukin-1beta receptor suppresses the voltage-gated potassium currents in the small-diameter trigeminal ganglion neurons following peripheral inflammation Pain 2008 139 594 602 10.1016/j.pain.2008.06.015 18694623

[B52] Hazuda DJ Strickler J Kueppers F Simon PL Young PR Processing of precursor interleukin 1 beta and inflammatory disease J Biol Chem 1990 265 6318 6322 2156847

[B53] Schonbeck U Mach F Libby P Generation of biologically active IL-1 beta by matrix metalloproteinases: a novel caspase-1-independent pathway of IL-1 beta processing J Immunol 1998 161 3340 3346 9759850

[B54] Parks WC Wilson CL Lopez-Boado YS Matrix metalloproteinases as modulators of inflammation and innate immunity Nat Rev Immunol 2004 4 617 629 10.1038/nri1418 15286728

[B55] Fantuzzi G Ku G Harding MW Livingston DJ Sipe JD Kuida K Response to local inflammation of IL-1 beta-converting enzyme- deficient mice J Immunol 1997 158 1818 1824 9029121

[B56] Ito A Mukaiyama A Itoh Y Nagase H Thogersen IB Enghild JJ Degradation of interleukin 1beta by matrix metalloproteinases J Biol Chem 1996 271 14657 14660 10.1074/jbc.271.25.14657 8663297

[B57] Chen L Huang LY Sustained potentiation of NMDA receptor-mediated glutamate responses through activation of protein kinase C by a mu opioid Neuron 1991 7 319 326 10.1016/0896-6273(91)90270-A 1678615

[B58] Drdla R Gassner M Gingl E Sandkuhler J Induction of synaptic long-term potentiation after opioid withdrawal Science 2009 325 207 210 10.1126/science.1171759 19590003

[B59] Zhou HY Chen SR Chen H Pan HL Opioid-induced long-term potentiation in the spinal cord is a presynaptic event J Neurosci 2010 30 4460 4466 10.1523/JNEUROSCI.5857-09.2010 20335482 PMC2852319

[B60] Ji RR Samad TA Jin SX Schmoll R Woolf CJ p38 MAPK activation by NGF in primary sensory neurons after inflammation increases TRPV1 levels and maintains heat hyperalgesia Neuron 2002 36 57 68 10.1016/S0896-6273(02)00908-X 12367506

[B61] Obata K Katsura H Mizushima T Yamanaka H Kobayashi K Dai Y TRPA1 induced in sensory neurons contributes to cold hyperalgesia after inflammation and nerve injury J Clin Invest 2005 115 2393 2401 10.1172/JCI25437 16110328 PMC1187934

[B62] Zhuang ZY Wen YR Zhang DR Borsello T Bonny C Strichartz GR A peptide c-Jun N-terminal kinase (JNK) inhibitor blocks mechanical allodynia after spinal nerve ligation: respective roles of JNK activation in primary sensory neurons and spinal astrocytes for neuropathic pain development and maintenance J Neurosci 2006 26 3551 3560 10.1523/JNEUROSCI.5290-05.2006 16571763 PMC6673862

[B63] Gold MS Weinreich D Kim CS Wang R Treanor J Porreca F Redistribution of Na(V)1.8 in uninjured axons enables neuropathic pain J Neurosci 2003 23 158 166 12514212 10.1523/JNEUROSCI.23-01-00158.2003 PMC6742156

[B64] Alessandri-Haber N Dina OA Chen X Levine JD TRPC1 and TRPC6 channels cooperate with TRPV4 to mediate mechanical hyperalgesia and nociceptor sensitization J Neurosci 2009 29 6217 6228 10.1523/JNEUROSCI.0893-09.2009 19439599 PMC2726836

[B65] Luo MC Zhang DQ Ma SW Huang YY Shuster SJ Porreca F An efficient intrathecal delivery of small interfering RNA to the spinal cord and peripheral neurons Mol Pain 2005 1 29 10.1186/1744-8069-1-29 16191203 PMC1253531

[B66] Mizushima T Obata K Yamanaka H Dai Y Fukuoka T Tokunaga A Activation of p38 MAPK in primary afferent neurons by noxious stimulation and its involvement in the development of thermal hyperalgesia Pain 2005 113 51 60 10.1016/j.pain.2004.09.038 15621364

[B67] Stone LS MacMillan LB Kitto KF Limbird LE Wilcox GL The alpha2a adrenergic receptor subtype mediates spinal analgesia evoked by alpha2 agonists and is necessary for spinal adrenergic-opioid synergy J Neurosci 1997 17 7157 7165 9278550 10.1523/JNEUROSCI.17-18-07157.1997 PMC6573259

[B68] Jin SX Zhuang ZY Woolf CJ Ji RR p38 mitogen-activated protein kinase is activated after a spinal nerve ligation in spinal cord microglia and dorsal root ganglion neurons and contributes to the generation of neuropathic pain J Neurosci 2003 23 4017 4022 12764087 10.1523/JNEUROSCI.23-10-04017.2003 PMC6741086

[B69] Zhuang ZY Gerner P Woolf CJ Ji RR ERK is sequentially activated in neurons, microglia, and astrocytes by spinal nerve ligation and contributes to mechanical allodynia in this neuropathic pain model Pain 2005 114 149 159 10.1016/j.pain.2004.12.022 15733640

[B70] Berta T Poirot O Pertin M Ji RR Kellenberger S Decosterd I Transcriptional and functional profiles of voltage-gated Na(+) channels in injured and non-injured DRG neurons in the SNI model of neuropathic pain Mol Cell Neurosci 2008 37 196 208 10.1016/j.mcn.2007.09.007 17964804

